# Barriers & Facilitators of Innovation in Long-Term Care Homes During COVID-19 in Brazil: views from key stakeholders

**DOI:** 10.1093/geroni/igaf122.3022

**Published:** 2025-12-31

**Authors:** Suzanne Santos, Gilciney Rabello, Gabriela Nacimben, Marisa Domingues, Meire Cachioni, Patrick Wachholz, Charlene Chu, Ruth Melo

**Affiliations:** Universidade de São Paulo, São Paulo, Sao Paulo, Brazil; Universidade de São Paulo, São Paulo, Sao Paulo, Brazil; Universidade de São Paulo, São Paulo, Sao Paulo, Brazil; Universidade de São Paulo, São Paulo, Sao Paulo, Brazil; Universidade de São Paulo, São Paulo, Sao Paulo, Brazil; Universidade Estadual Paulista, Botucatu, Sao Paulo, Brazil; University of Toronto, Toronto, Ontario, Canada; Universidade de São Paulo, São Paulo, Sao Paulo, Brazil

## Abstract

The COVID-19 pandemic had a significant effect on individuals residing and working in long-term care homes (LTCHs). This scenario highlighted systemic disadvantages and vulnerabilities and called for innovations in the sector around the world. In Brazil, the LTC segment is historically marked by inequalities, underfunding, absence of systematically organized data and research. Therefore, little is known about the perception of key stakeholders about innovation in LTC. This cross-sectional qualitative study, part of the CONNECTED consortium, investigates potential barriers and facilitators in implementing innovations during COVID-19 from the perspective of key stakeholders from four LTCHs. The team conducted 15 semi-structured interviews (residents: n = 5, family members/caregivers: n = 5, staff: n = 5) from March to October 2024. Interviews were transcribed verbatim and the results were analyzed using thematic analysis through Rapid Assessment Procedure (RAP) sheets. Besides financial constraints and workforce shortage, barriers to innovation during COVID-19 included lack of involvement in decision-making for residents, family members, and direct care workers. Residents reported uncertainty in whether their opinions were considered. Communication issues were shared by all groups and influenced their ability to adapt to the new protocols. Facilitators included the willingness to change and the perceived gains of innovations, including enhanced safety measures and the use of technology, which was critical for staying connected. Staff emphasized the need for structured support, while families focused on emotional connections. Such insights underscore the need for inclusive, co-designed practices in Brazilian LTCHs in order to empower the sector and ensure the dignity of such populations.

